# Design, Fabrication, and Surgical Testing of the 3D-Printed Large-Array Port-System for the Implantation of Large Epiretinal Stimulators

**DOI:** 10.1167/tvst.14.2.8

**Published:** 2025-02-05

**Authors:** Frederic Kuba Balcewicz, Sabine Baumgarten, Kim Schaffrath, Jiayun Wang, Sandra Johnen, Peter Walter, Tibor Lohmann

**Affiliations:** 1 Department of Ophthalmology, RWTH Aachen University, Aachen, Germany

**Keywords:** retinal implants, retinal prostheses, ophthalmologic surgery, implantation device, port system, 3D-printing, medical engineering, vitreoretinal surgery

## Abstract

**Purpose:**

In the treatment of blindness causing retinal dystrophies, that is, retinitis pigmentosa (RP), retinal implants showed promising results. Recently, larger devices restoring a greater visual field were introduced. With larger size, implantation surgery became more difficult. In this study, a novel implantation device was developed, fabricated, and tested in implantation surgeries. The goal was to demonstrate a reproducible, safe, and, in comparison, superior implantation method.

**Methods:**

The novel implantation device 3D-Printed Large-Array Port-System (3D-PLAPS) was designed using computer-aided design software. Anatomic dimensions of rabbit, pig, and human eyes were collected from anatomic and histological data sources. The 3D-PLAPS were 3D-printed. In cadaveric porcine and rabbit eyes, 3D-PLAPS was used to implant large epiretinal stimulators developed by this group. A standardized surgical procedure was established. Intraocular pressure (IOP) was measured.

**Results:**

The 3D-PLAPS implantation device was designed with a length of 8.4 mm and adapted to the curvature of normal sighted human eyes with a diameter of 24.0 mm. The elliptical aperture is 7.0 mm in length and 1.0 mm in width at its widest points. Marginal apertures for scleral fixation were added. A closing plug was introduced. Design and dimensions were adapted for rabbit eyes. During surgery, the 3D-PLAPS improved ocular stability, sealed the incision, and withstood an elevated IOP. It was suitable for foldable stimulators with a diameter of up to 14.0 mm.

**Conclusions:**

The 3D-PLAPS implantation device showed feasibility in implantation of large epiretinal stimulators and possibly also facilitates repositioning of stimulating arrays in acute experiments without the necessity for additional surgical steps.

**Translational Relevance:**

The 3D printing and CAD software are used to applied surgery for large epiretinal stimulators.

## Introduction

Retinal dystrophies, that is, retinitis pigmentosa (RP), are progressive diseases affecting the retinal pigment epithelium (RPE) and photoreceptors.^1^^,^^2^ RP is caused by various mutations coding for enzymes involved in retinal metabolism and light perception.^3^ RP affects approximately 1 in 5000 individuals worldwide.^4^ After onset nyctalopia, a progressive loss of visual field follows. In RP, preservation of inner retinal neurons can be seen, despite a nearly total loss of photoreceptors.^4^ Neurons, to which photoreceptors connect, partially survive.^5^^–^^8^ Currently, treatment options are limited with different treatments under evaluation: nutritional supplements, pharmaceutical therapies, retinal implants, gene therapy, and stem cell approaches.^2^^,^^9^^,^^10^ In previous clinical studies, it was shown that electrical retinal stimulation applied by microelectrodes can initiate a local neural response, leading to visual perception in clinically blind patients.^11^ Retinal stimulators have been used in patients with RP.^12^ With the introduction of the Argus II (Second Sight Medical Products, Sylmar, CA, USA), retinal stimulators were made commercially available to a larger group of clinicians and patients.^13^^,^^14^ For blind patients with RP, the re-activation of a larger visual field is important to regain mobility and orientation. Therefore, large-area retinal stimulators are the focus in current research.^1^^,^^15^^,^^16^ As published in 2019 by our group, a very large electrode array (VLARS) was introduced to obtain a large visual angle with 250 electrodes on a foldable epiretinal array.^15^ Further developments toward a larger area of stimulation followed.^16^^–^^18^

Generally, the implantation of larger arrays results in an increasingly more traumatic surgery.^15^ The demand for larger incisions yields a higher risk for intravitreal hemorrhage, retinal tearing and detachment, implant displacement due to uncontrolled intraocular flow, leakage and hypotonia, and proliferative tissue reactions.^1^^,^^10^^,^^15^^,^^17^^,^^19^ Results on the implantation surgery published by this group showed feasibility, yet adverse events, that is, retinal detachment or hemorrhage, occurred.^15^^,^^17^ Therefore, the introduction of a safer and more efficient surgery method for large-area electrode arrays is desired. Further, the possibility to reposition the implanted arrays in an acute setting is desirable.

In this study, we designed, produced, and surgically tested an implantation device for large epiretinal stimulators. The 3D-Printed Large-Array Port-System (3D-PLAPS) was tested in cadaveric porcine and rabbit eyes for its surgical feasibility. Further, intraocular pressure (IOP) while using the 3D-PLAPS was evaluated. The aim was to create an implantation device which facilitates a safer surgery with stable IOP, reduced leakage, and the possibility to adjust the position of epiretinal stimulators during surgery, without the need for additional incisions or sutures.

## Materials and Methods

### Fabrication

#### Computer-Aided Design

To design the devices, 3D computer-aided design software AutoCAD 2022, Inventor 2022, and Fusion360 2022 (Autodesk, San Rafael, CA, USA) were used.

##### Dimensions of 3D-PLAPS Three-Part System

**Figure 1.**
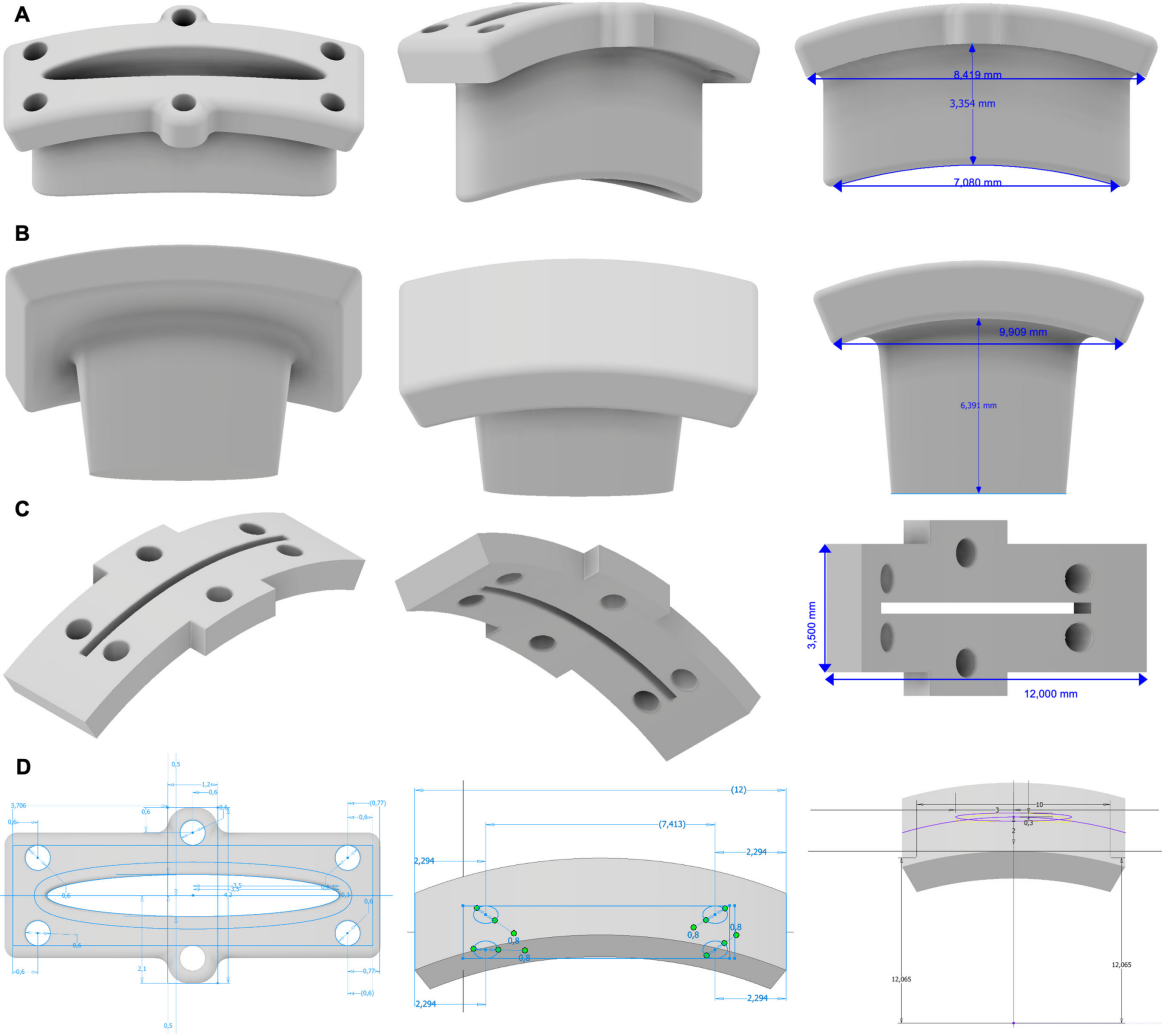
The 3D-PLAPS implantation device, closing plug, and pre-implantation template dimensions as 3D pictures in computer-aided design software Inventor 2022 (Autodesk, Rehovot, Israel); 3D-PLAPS oblique front, side-view with intraocular reaching elliptic tube, elliptic aperture width (1.0 mm), length (7.1 mm), complete length (8.4 mm), and intraocular depth (3.4 mm) (**A**); 3D-PLAPS closing plug depth (6.4 mm) and length (9.9 mm) (**B**); pre-implantation template length (12.0 mm) and width (3.5 mm) (**C**); 3D-PLAPS design in computer-aided software Inventor; 3D-PLAPS, pre-implantation template, and closing plug measurements (**D**) during the design and fabrication processes.

The 3D-PLAPS was designed in two planes: (1) a rectangular support frame to be sewed onto the sclera, and (2) an intraocular elliptical tube with a depth of 3.4 mm. The 3D-PLAPS was designed with a length of 8.4 mm and width of 3.0 mm. The elliptical aperture is measuring 7.0 mm in diameter and an opening width of 1.0 mm at its widest point. The aperture's wall thickness is 0.3 mm (Figs. 1A, 1D). For additional stability, the device is adapted to the curvature of normal sighted human eyes with an axial length (AL) of 24.0 mm. Apertures for scleral suturing were added to the base of the device (see Fig. 1A). To secure appropriate intraoperative stability, 6 marginal apertures were added, 0.6 mm in diameter each. The 3D-PLAPS can be occluded using a custom-made closing plug (see Figs. 1B, 1D). For correct surgical positioning of the 3D-PLAPS, a pre-implantation template was designed (see Figs. 1C, 1D). In summary, the 3D-PLAPS consists of three parts: a pre-implantation template, the device itself, and a closing plug (dimensions shown in the Table). For in vivo rabbit experiments, we adapted the design and dimensions of the 3D-PLAPS to the smaller rabbit eye with an AL of 16.0 mm with a more curved scleral base. Total length was shortened to 4.2 mm, width was shortened to 2.0 mm. The aperture's shape was changed to rectangular with a 2.5 × 1.5 mm opening. Suture holes have been reduced from six to two holes closer to the opening (see the Table). The intraocular part has been shortened to 1.5 mm in respect to the relative size comparing rabbit to human or porcine eyes.^20^

**Table.** Dimensions and Measurements of 3D-PLAPS Three-Part-Component System, in Millimeters

Implant	Length	Width	Depth	Aperture	Suture Holes	Holes Diameter
3D-PLAPS implantation device	8.4	3.0	3.4	7.1 × 1.0	6	0.6
3D-PLAPS implantation device for rabbit eyes	4.2	2.0	1.5	2.5 × 1.5	2	0.6
3D-PLAPS pre-implantation template	12.0	3.5 – 4.8	–	8.7 × 0.3	6	0.8
3D-PLAPS pre-implantation template for rabbit eyes	4.2	2.5	–	2.5 × 0.35	2	0.8
3D-PLAPS closing plug	9.9	5.0	6.4	–	–	–
3D-PLAPS closing plug for rabbit eyes	5.2	3.0	2.7	–	–	–

**Figure 2.**
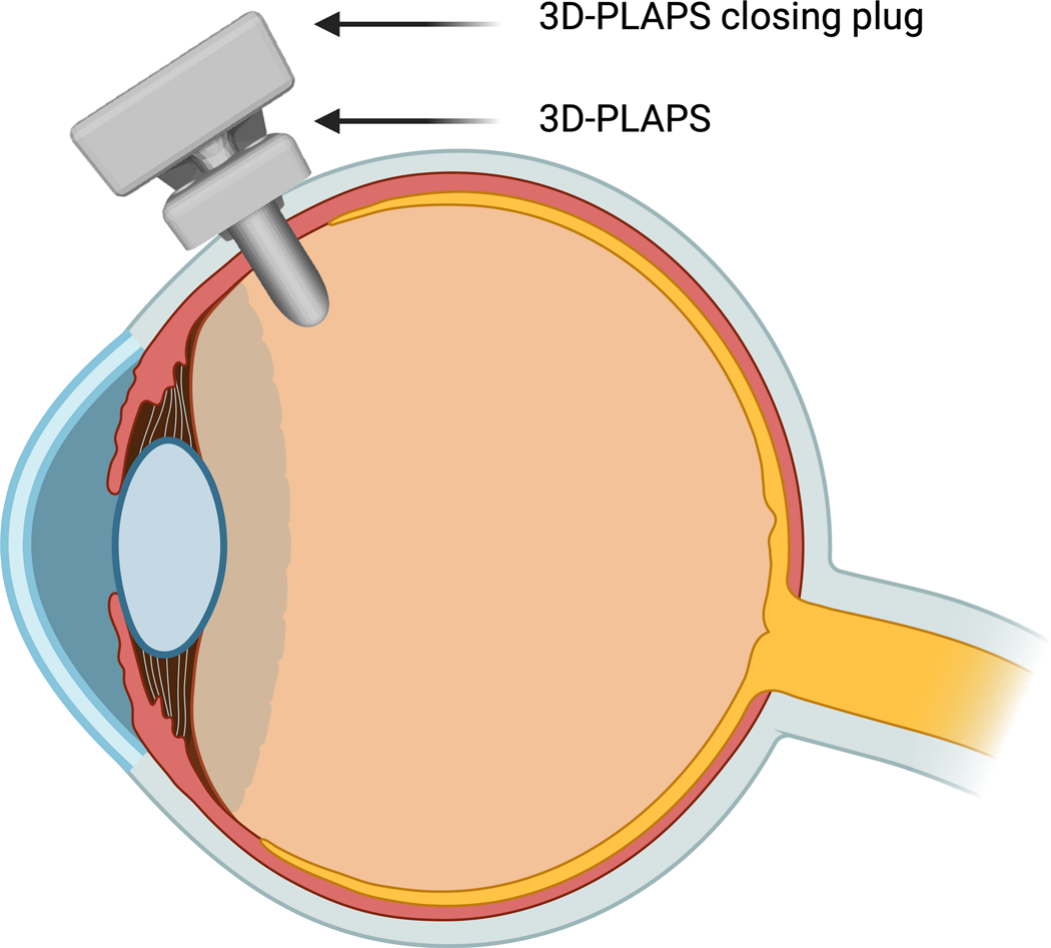
Schematic sagittal illustration of 3D-PLAPS and closing plug aligned at the pars plana of a human eye, 3D-PLAPS in closed stage after insertion; Created in BioRender. Balcewicz, F. (2024), https://BioRender.com/k06s246.

Figure 2 displays a schematic sagittal illustration of a human eye implanted with the 3D-PLAPS and 3D-PLAPS closing plug at the pars plana.

#### Three Dimensional Printing

The 3D-printing was performed using the Objet350 and Objet500 Connex 3 systems (Stratasys, Rehovot, Israel) at the Center for Biohybrid Medical Systems (CBMS) in Aachen, Germany (Institute of Applied Medical Engineering, RWTH University Aachen, Aachen, Germany). In its initial iteration, the 3D-PLAPS was fabricated using *HighTemperatureWhite* 3D-printing material (SDS-06109 EN A, RGD525; Stratasys, Rehovot, Israel). *RGD810* (VeroClear; Stratasys, Rehovot, Israel) was utilized for later iterations. Printed structures needed to be cleaned with mechanical instruments (Geuder AG, Heidelberg, Germany) and 70.0% ethanol to minimize residues.

### Cadaveric Eye Experiments

The surgical feasibility and device handling was tested in porcine and rabbit eyes in a similar setting to human ophthalmologic surgeries. All animal experiments were performed according to the ARVO declaration for the use of animals in research and adhered to the “Principles of laboratory animal care” (NIH publication No. 85-23, revised 1985), the OPRR Public Health Service Policy on the Human Care and Use of Laboratory Animals (revised 1986), and the US Animal Welfare Act, as well as according to the German Law for the Protection of Animals and after obtaining approval by local authorities and ethics committee.

Surgeries were performed using a surgical microscope (Zeiss OPMI MDO S5 Ophthalmology Surgical Microscope; Carl Zeiss Meditech, Oberkochen, Germany) by an experienced retinal surgeon (author T.L.) using a phacoemulsification and vitrectomy device (Fritz Ruck Pentasys 1 Phaco Emulsifier, Fritz Ruck Ophthalmologische Systeme; GmbH, Eschweiler, Germany).

**Figure 3.**
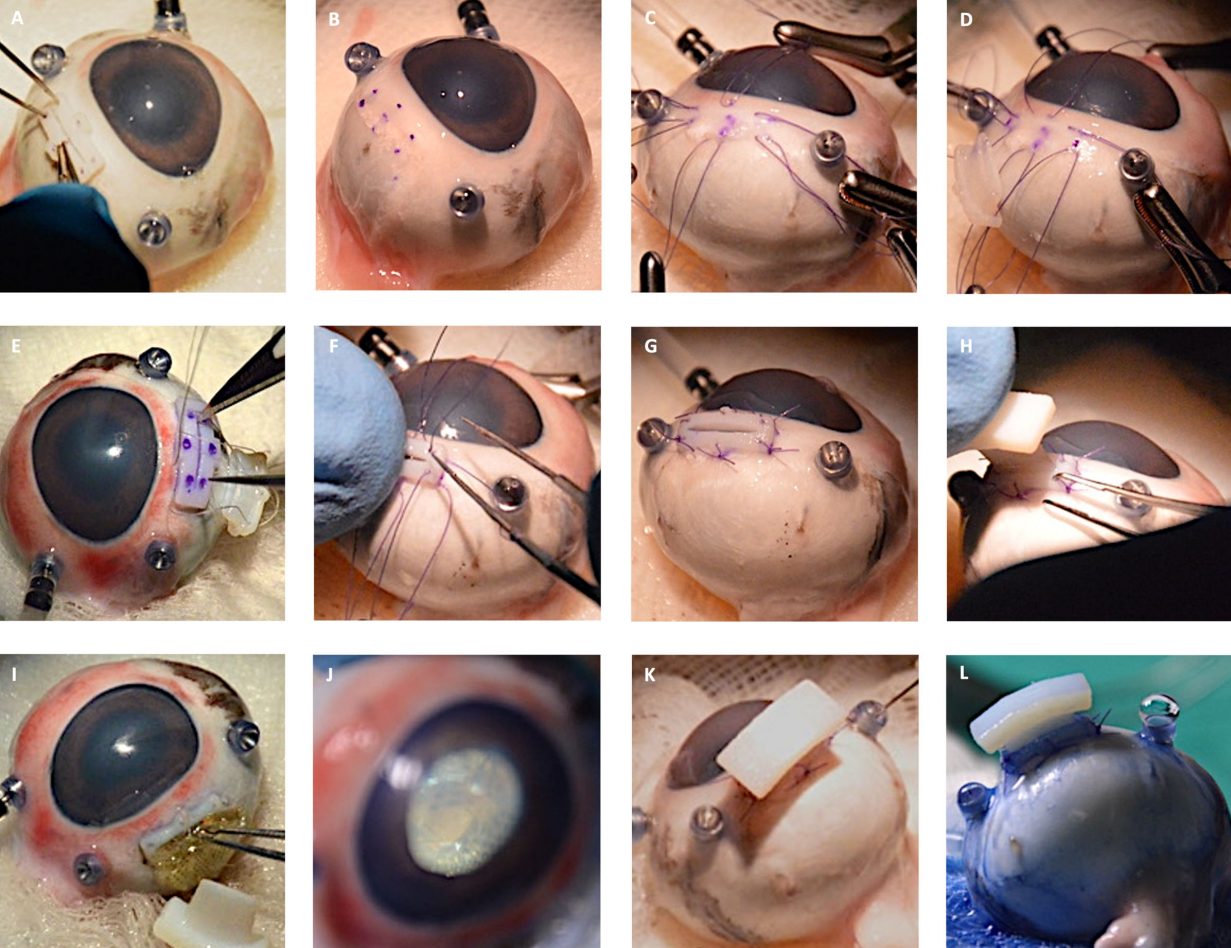
Porcine eye. Ophthalmological vitrectomy setup consisting of three 23G trocars (EVA AVETA23G Cannula set with high flow infusion line, Article No: 1272.ED23, D.O.R.C. Dutch Ophthalmic Research Center (International) B.V., Zuidland, The Netherlands), running infusion, marking of 6 suture points with surgical marker using 3D-PLAPS pre-implantation template (**A**, marked in **B**), suturing process with 6-0 Vicryl surgical suture (ETHICON; Johnson & Johnson, New Brunswick, NJ, USA) and stabilized by 6 Dieffenbach serrefine forceps (Geuder AG, Heidelberg, Germany) (**C**), attachment of 3D-PLAPS device onto 3 bottom sutures (**D**), use of pre-implantation template for marking of incision points, scalpel (size 11) incision (Feather disposable scalpel, Feather Safety Razor Co., LTD., Osaka, Japan, Socorex Isba SA, Ecublens/Lausanne, Switzerland) (**E**), scleral fixation of 3D-PLAPS with 6-0 Vicryl surgical sutures (**F**), result of attachment (**G**), adapting the closing plug to the device (**H**), implantation of VLARS epiretinal stimulator dummy through 3D-PLAPS and correct positioning inside eye cavity epiretinally (**I, J**), trypan blue tests for sealing and leakage (Vioron Vial 0.5 mL, G-81001, 1000188, Fluoron GmbH, Ulm, Germany), semi-quantitative flow analysis inside eye (**K, L**) (showing the closing plug on 3D-PLAPS with leakage of the infusion trough 23G trocar system).

**Figure 4.**
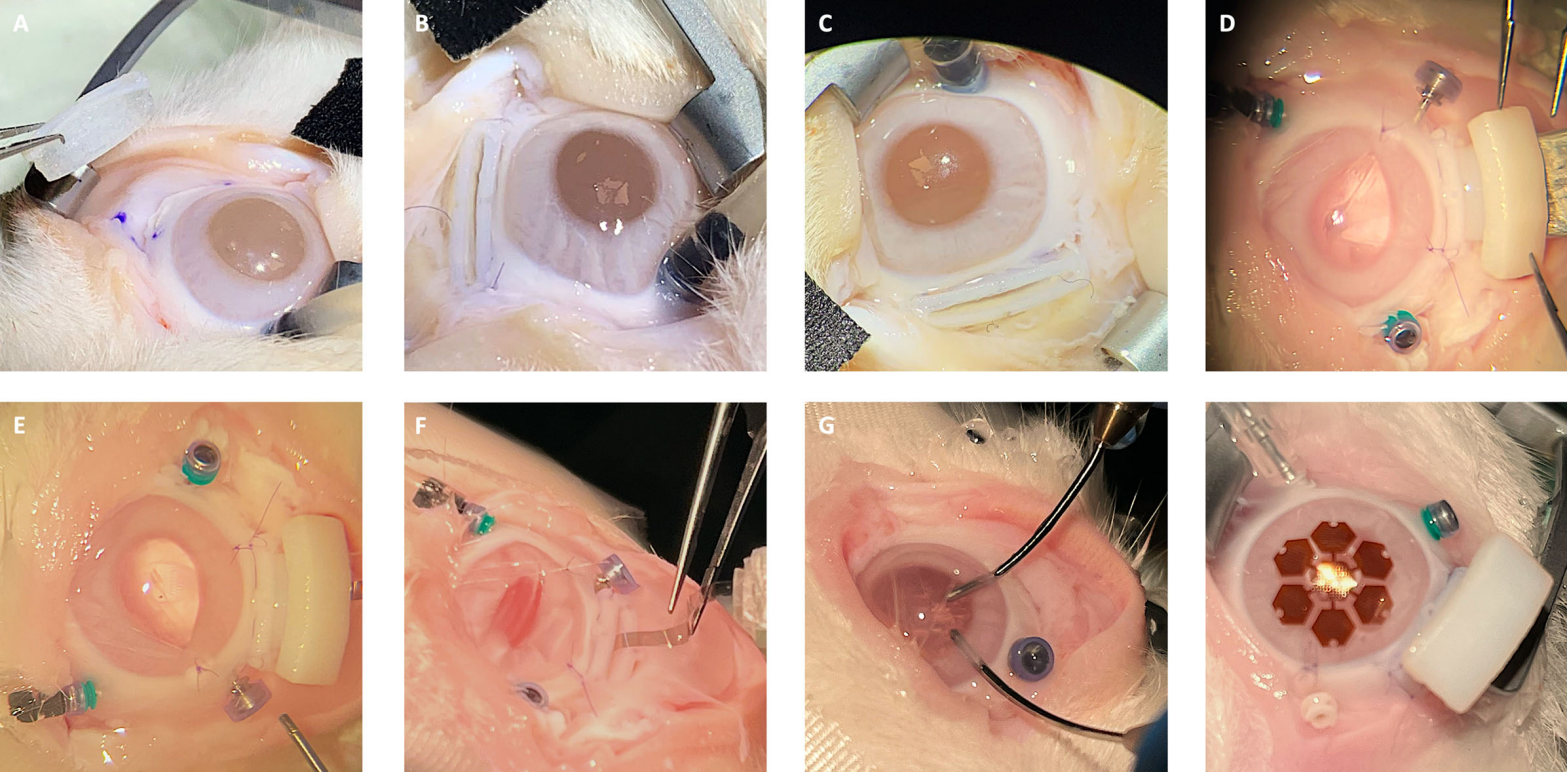
Albino rabbit eye in its orbit. Insertion of 3D-PLAPS using a micro colibri forceps and Cook eye speculum (Geuder AG, Heidelberg, Germany) (**A**), fixation using ophthalmological surgery sutures (**B**), inserted into scleral incision, and fixated under a microscope (**C**), 23G vitrectomy trocar set (EVA AVETA 23G Cannula set with high flow infusion line, Article No: 1272.ED23, D.O.R.C. Dutch Ophthalmic Research Center (International) B.V., Zuidland, The Netherlands), fixated and closed using 3D-PLAPS closing plug (**D**), insertion experiments (**D–F**), irrigation and aspiration (**G**), mounded 3D-PLAPS, novel developed DEARS (**E**) (Ghosh et al., 2024) lying on the cornea, pressured eye (**H**).

**Figure 5.**
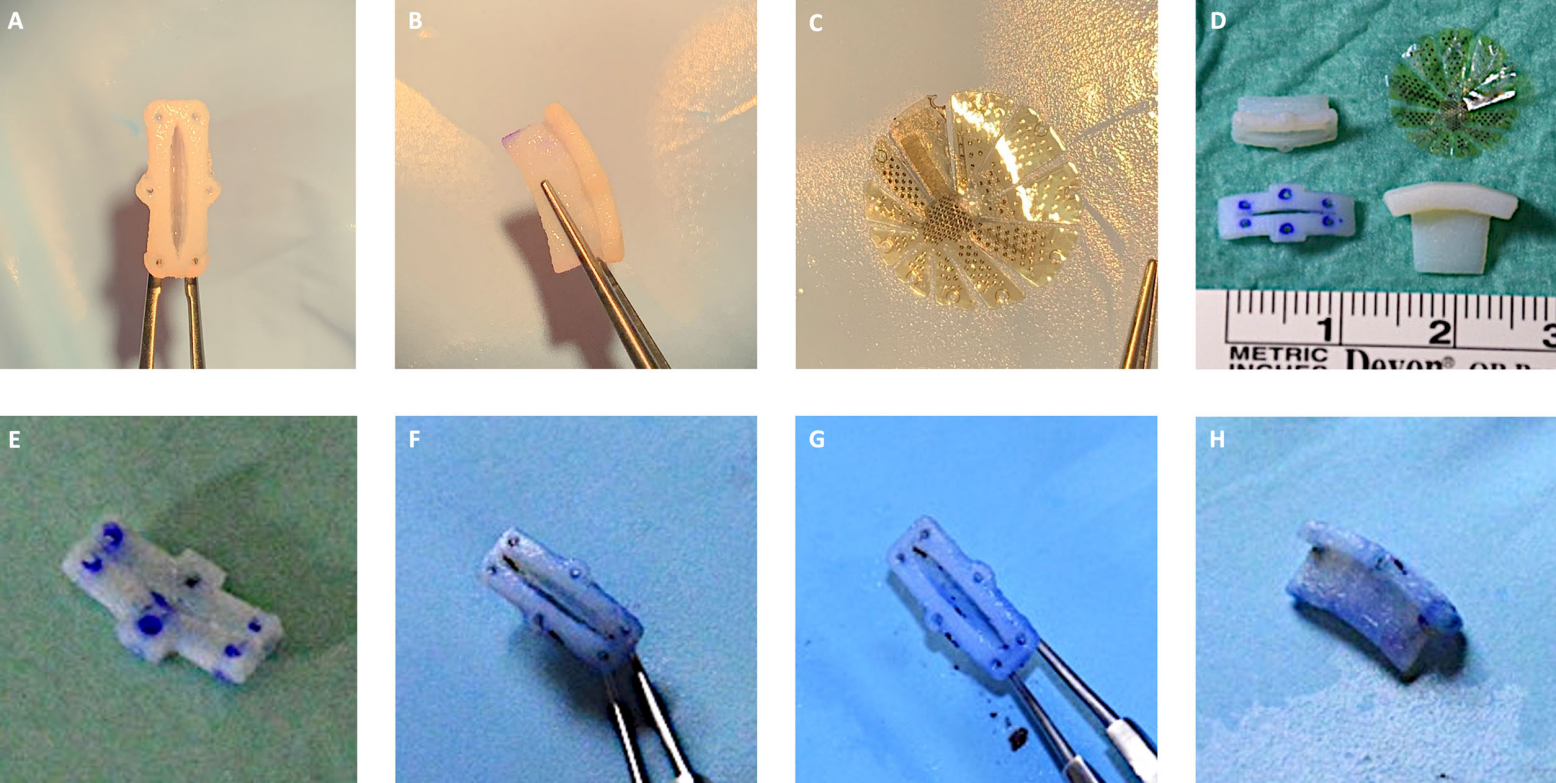
Porcine cadaveric eye, 3D-PLAPS post-surgery: *upper view* (**A**), *side view* (**B**), VLARS dummy from top (**C**), scale of all used 3D-PLAPS – instruments and VLARS (**D**), 3D-PLAPS and pre-implantation template post-surgery, upper view of the template (**E**), upper view of the 3D-PLAPS (**F, G**), side view of the 3D-PLAPS (**H**); visible blue coloring comes from surgical marker and trypan blue leakage and sealing measurements.

Porcine cadaveric eyes were stabilized in a custom-made 3D-printed holding device. We performed a corneal abrasion on selected porcine eyes using a Szurman DMEK Preparation Scraper (Geuder, Heidelberg, Germany) in case of advanced epithelial edema for better visibility. Additionally, Glycerin 0.25% eye drops (Pharmacy of the RWTH University Hospital, Aachen, Germany) were used on the corneal surface to further reduce opacity. Three 23-gauge trocars (EVA AVETA 23G Cannula set with high flow infusion line, D.O.R.C. Dutch Ophthalmic Research Center International B.V., Zuidland, The Netherlands) for infusion, a cutter (High Speed vitrector Cutter UNO Colorline 23G; Geuder AG, Heidelberg, Germany) and a light source (fiber optic S UNO Colorline 23G; Geuder AG, Heidelberg, Germany) were set 3.5 mm distally from the corneal limbus in the following positions: nasal superior, temporal superior, and temporal inferior. A balanced salt solution infusion was connected to maintain a constant IOP. The lens was removed by phacoemulsification followed by complete vitrectomy (Fritz Ruck Pentasys 1 Phaco Emulsifier). The 3D-PLAPS pre-implantation template was placed episclerally between two 23G trocar ports, approximately 3.5 mm distally from the corneal limbus (Fig. 3A). Six suture points were marked (Fig. 3B). The template was removed, and 6 intrascleral 6-0 Vicryl monophilic seams (ETHICON; Johnson & Johnson, New Brunswick, NJ, USA) were placed (Figs. 3B–D). The 3D-PLAPS template was placed episclerally, an 8.7 mm incision was performed using a disposable scalpel (Feather disposable scalpel number 11; Feather Safety Razor Co., LTD., Osaka, Japan; Fig. 3E). After the template was removed, the 3D-PLAPS was inserted (see Figs. 3E, 3F). The base of the 3D-PLAPS was sutured onto the sclera (Fig. 3G) and the 3D-PLAPS closing plug was inserted (Fig. 3H). Handling was tested with the implantation of the VLARS and a novel epiretinal stimulator (Dense Electrode Array for Retinal Stimulation [DEARS]) developed by our group (unpublished data, E. Ghosh et al., 2024; Figs. 3I, 3J).^15^ The surgery in rabbits followed the steps as described above except the 23-gauge trocars were placed approximately 2.0 mm distally of the limbus. The surgery was conducted on decapitated heads rather than enucleated eyes (Figs. 4A–G). Design and dimensions were kept the same as for the porcine eyes in the first iterations. Those designs can be seen in Figures 4A to 4C. Figures 4D to 4H display 3D-PLAPS in its design for porcine experiments. In later experiments, the dimensions of the 3D-PLAPS were changed and a novel design was established (see the Table). Surgery steps are shown in Figures 4A to 4C. Insertion experiments are displayed in Figures 4D to 4F. Post-surgery images of the 3D-PLAPS, the 3D-PLAPS pre-implantation template, the 3D-PLAPS closing plug, and the implanted epiretinal stimulator were taken to evaluate possible signs of damage or utilization (Fig. 5).

### Pressure Measurements

**Figure 6.**
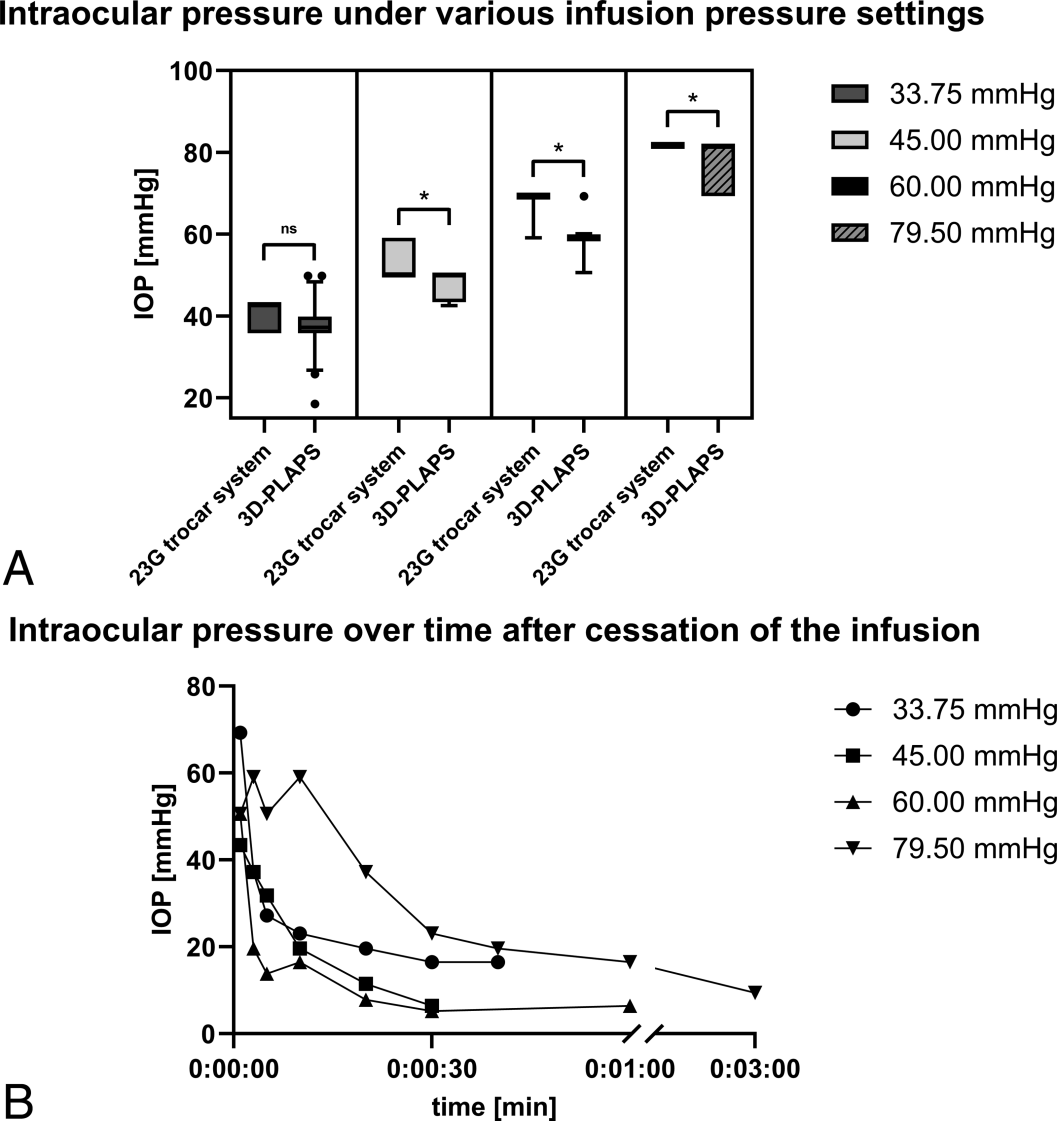
Pressure experiments with 23G trocar system both with and without additional implementation of 3D-PLAPS. (**A**) Results of intraocular pressure (IOP) measurements with running infusion under various infusion pressure settings, with the 3D-PLAPS closed using its closing plug. (**B**) Results of IOP stability over time after cessation of the infusion, with the 3D-PLAPS closed using its closing plug. (**A**) The graph shows an analysis of resulting IOP levels obtained using either a 23G trocar port system alone or in conjunction with the 3D-PLAPS in a closed configuration. The measurements were conducted using a Schioetz tonometer at four distinct pressure intervals (33.75 mm Hg, 45.00 mm Hg, 60.00 mm Hg, and 79.50 mm Hg). At each pressure interval, five pressure data points were analyzed. The results were compared to the corresponding pressure data gained without the setup of 3D-PLAPS. Bars were calculated as mean ± SD. Whiskers represent the 10th and 90th percentiles. Maxima and minima are displayed as *black dots*. Ordinary 1-way ANOVA (Dunnett’s multiple comparison test) was used. A *P* value of < 0.05 was considered statistically significant. Significance is marked an asterisk (*), not significant differences is marked with “ns.” (**B**) The graph shows the timespan of IOP values after cessation of the infusion (0:00:00 seconds). IOP measurements were recorded using a Schioetz tonometer at time intervals of 1 second, 3 seconds, 5 seconds, 10 seconds, 20 seconds, 30 seconds, 40 seconds, 60 seconds, and 180 seconds. For each set infusion pressure, the same IOP-measurement method was performed.

Indentation tonometry was performed with a Schiötz type tonometer (Geuder AG, Heidelberg, Germany) in the closed and opened states of the 3D-PLAPS. Measurements were conducted with the infusion turned on and off (Fig. 6). The IOP was set to 33.75, 45.00, 60.00, and 79.50 millimeters of mercury (mm Hg) over the infusion, followed by repeated (5 times) IOP measurements for each step. The objective was to confirm the absence of leakage and the maintenance of IOP. Trypan Blue (Vioron Vial 0.5 mL, G-81001, 1000188; Fluoron GmbH, Ulm, Germany) was intravitreally injected to visualize leakage (Figs. 3K, 3L).

### Statistical Analysis

For continuous measures, the ordinary 1-way ANOVA (Dunnett’s multiple comparison test) was used. A *P* value of < 0.05 was considered statistically significant. All statistical analyses were performed with GraphPad Prism (GraphPad Prism 10, San Diego, CA, USA).

### Sterilization

Sterilization was performed using propanol-2-propanol (Merck KGaA, Darmstadt, Germany), 70.0% ethanol (Otto Fischar GmbH & Co. KG, Saarbrücken, Germany), and autoclave (Systec DX-23 horizontal bench-top autoclave; Systec GmbH & Co. KG, Linden, Germany) for packaged rubber/plastics (121°C/20 minutes, total duration of 37 minutes) at the Interdisciplinary Center for Clinical Research (IZKF, University Hospital RWTH Aachen, Aachen, Germany).

## Results

### Design

The dimensions of the 3D-PLAPS were adapted to human eyes with an AL of 24.0 mm, first both for experiments on cadaveric rabbit and porcine eyes. The axisymmetric design proved to be suitable for the implantation of large, flat objects. The 3D-PLAPS was designed with its currently used dimensions, as seen in the Table, based on the dimension of VLARS, OPTO-EPIRET, and the newly developed epiretinal stimulator DEARS (Fig. 4H).^15^^,^^17^ The adaptation of 3D-PLAPS’ dimensions to rabbit eyes, as described before, still allowed the array and the implantation forceps to be inserted (see Figs. 3I, 3J).

In early iterations of the 3D-PLAPS, hollow walls and insufficient stability caused difficulties as the structures were easily damaged on light touch during preliminary implantation experiments. In later iterations, the Inventor rather than AutoCAD was used, resulting in a more comprehensive design process. This resulted in an increase in stability while using the same printing material. To further improve stability, a curved base plate was added in the following designs. Seam material up to a thread thickness of 3-0 was successfully used.

As the open 3D-PLAPS caused leakage and hypotonia, a closing mechanism was added to ensure ocular stability after implanting. For the closing plug, we also chose a 3D-printed approach. The closing plug is designed with a tapered shape to completely close the aperture (see Fig. 1B). During surgical experiments, a pre-implantation template was considered to reduce surgery time (see Fig. 1C).

### Three-dimensional Printing Method and Material Selection

Initially, the 3D-PLAPS was printed utilizing *RGD525* 3D-printing material. It has suitable dimensional stability and heat resistance.^21^^,^^22^ The printing method was feasible with no complications observed. Structures printed with RGD525 were fabricated with the support material SUP705. No issues occurred during the cleaning processes. *RGD810* (VeroClear; Stratasys, Rehovot, Israel) was used for later iterations due to more transparency and a nearly colorless material, because enhanced optical clarity can be advantageous for medical applications.^23^ Printing of both RGD810 and RGD525 was completed in a time span of approximately 2 hours. We produced 10 units of the 3D-PLAPS, pre-implantation template, and closing plug structure in 1 roll, and a total of 50 of the 3D-printed structures each. The cleaning processes of RGD525 and RGD810 were comparable with approximately 3 minutes for each device.

### Implantation Surgery

#### Porcine Implantation Surgery

Surgeries in cadaveric eyes of pigs demonstrated the feasibility of a transscleral surgical approach for inserting the 3D-PLAPS. The insertion was easiest if 3D-PLAPS was positioned as seen in Figure 3. The surgeon rated the procedure as complex but feasible and safe. A total of 14 surgeries were performed on 14 cadaveric porcine eyes. Surgery duration decreased over the course of the surgeries, with an average duration of approximately 60 minutes. Lensectomy and pars-plana vitrectomy were conducted in suitable durations and without difficulties. Insufficient suturing and an overly wide incision caused low IOP in early experiments but was later solved by using 3D-PLAPS pre-implantation template and the closing plug. Retinal detachments were detected in several cadaveric porcine eyes prior to surgery.

#### Rabbit Implantation Surgery

In the eight implantation surgeries in rabbit eyes, lensectomy and vitrectomy were performed without complications. Due to the smaller size of rabbit eyes, lack of space for three trocar ports and the 3D-PLAPS was a surgical challenge. In later experiments, 3D-PLAPS’ design was adjusted for rabbit eyes as seen in the Table. Retinal detachment did not occur before or after the performed surgeries.

#### Epiretinal Stimulator Implantation

In the cadaveric eye experiments, the implantation of an epiretinal stimulator developed by this group was evaluated.^15^^,^^17^ The VLARS structures were implanted without damage to the stimulator. Intraocular unfolding was performed directly after insertion with the help of ophthalmologic surgery instruments (see Figs. 3I, 3J). After inserting the VLARS structure, the bulb was tamponated with filtered air at an IOP of 25.0 mm Hg. All incisions (the ports and the scleral incision for 3D-PLAPS) were sutured. The novel epiretinal stimulator DEARS was implanted only in experiments with rabbit eyes, because future in vivo experiments will also be conducted in rabbits. Despite an incision of 4.0 mm, we achieved IOP stability while granting the possibility for intraocular implant manipulation. Overall, the 3D-PLAPS is suitable for large-area devices up to 14.0 mm in foldable and up to 7.0 mm in non-foldable states.

### Pressure Measurements

It was observed that in high IOP (> 74.0 mm Hg) Trypan-blue leaked through all three 23G trocar ports, but not past the closed 3D-PLAPS. Figure 6 shows the IOP over time in porcine eyes. The findings were evaluated in relation to the corresponding pressure data obtained from experiments conducted with three 23G trocar ports, in the absence of the 3D-PLAPS implanted.

With a running infusion, in eyes with the inserted 3D-PLAPS and closing plug, IOP approached the set pressure of the infusion (see Fig. 6A). Yet IOP was significantly lower at all measured infusion pressures except 33.75 mm Hg (see Fig. 6A). Although the IOP was lower in eyes with the 3D-PLAPS implanted, high IOPs close to those in eyes without the 3D-PLAPS were achieved (see Fig. 6A). Further, following cessation of infusion, the 3D-PLAPS can maintain IOP for a brief period (see Fig. 6B). Except for the set pressure of 79.50 mm Hg, all the other 3 pressure settings dropped within 60 seconds down to a non-quantifiable IOP (see Fig. 6B).

### Sterilization

Sterilization with the Systec DX-23 horizontal bench-top autoclave took 37 minutes. The 3D-printing material RGD525 remained undamaged. Methods using ethanol and propanol-2-propanol sterilization were not pursued further.

## Discussion

### Design and Materials

The implantation of epiretinal structures using the 3D-PLAPS three-part-system in cadaveric porcine and rabbit eyes had the purpose to establish the feasibility of the surgical procedure, especially for the later use in the in vivo experiments.

In the wake of new, larger, and more versatile retinal stimulators, there is a demand to improve the implantation into human eyes. In the early days of artificial vision, acute stimulation experiments were conducted on blind patients suffering from RP with the aim to elicit visual perceptions.^24^ Hornig et al. showed that it is possible to elicit electrically evoked phosphenes and to measure the perceptual threshold.^11^ In acute experiments, once the stimulating array was placed, repositioning was difficult.^11^^,^^15^ By using the 3D-PLAPS, the possibility for a repeated repositioning of the stimulating array is granted. For future applications of (epi)retinal stimulators, that is, penetrating arrays or bidirectional stimulators, a return to acute experiments in humans might be necessary. Here, the 3D-PLAPS can function as a helpful implantation tool. Regarding chronic stimulation experiments, stimulators may need to be repositioned during the initial implantation after establishing their retinotopic placement. To secure proper retinal alignment, measuring electrode impedance changes was successfully used in the past.^25^ In bi-directional stimulation approaches, measuring local neural activity can secure the retinal position.^26^ In cases where these approaches show an improper electrode placement, the 3D-PLAPS aids a successful repositioning in the initial implantation.

Initially, a general design of the implantation device was created, as an implantation of a uniform 3D-PLAPS in different sized eyes of various animal species was planned. Whereas an implantation of the initial 3D-PLAPS was possible in rabbit eyes, for in vivo experiments, a smaller version seemed more feasible. Swiftly changing the initial design was possible because of computer-aided design tools and 3D-printing. In future projects, we can adapt the 3D-PLAPS dimensions not only to different eye sizes, but also to the size of the implanted stimulating array. Overall, the process of redesigning, producing, and use in surgery demonstrated the possibilities of current technology in applied medicine.

The 3D-PLAPS was designed axisymmetric rather than rotationally symmetric, as observed in vitrectomy trocar systems. In line with commonly used trocars, we chose an intraocular depth of 3.4 mm to minimize possible traumatic damage to intraocular structures. The incidence of intraocular damage is expected to be reduced to a level associated with 23-gauge trocars due to the adjustment to align with the dimensions of 23-gauge trocar systems. The design is used in porcine eye experiments and fulfills the requirement of anatomic dimensions for human translation. To minimize the contact area between sclera and 3D-PLAPS, a pre-curved design of the 3D-PLAPS supports a better alignment. During experiments, a pre-implantation template was used for easier and reproducible intraoperative handling, a closing plug was necessary to stabilize the IOP. The established combination of a three-part port system yielded the best outcome in the conducted experiments.

For our purpose, the 3D-printing material needed to be high-quality, resistant to different fluids, simple in cleaning, and suitable for sterilization. Further, a short production time helped to quickly identify and consecutively change flaws in design and production. With both 3D-printers used, these goals were achieved.^27^ For future projects, it is recommended to transition from the currently used VeroClear 3D-printing material to Biocompatibility Clear MED610 (Stratasys, Rehovot, Israel) for improved biocompatibility. The proven biocompatibility of the 3D-printed material reduces the risk for intraocular inflammation.

An alternative production approach involved selective laser sintering (SLS) with EOS P110 Formiga (EOS, Krailling, Germany) at the Fraunhofer Institute for Laser Technology ILT (Additive Manufacturing, Fraunhofer ILT, Aachen, Germany) with Polyamide 12 (PA12) material (Marl Chemical Park, Marl, Germany). Production duration for a single SLS produced structure was 8 to 10 hours. Due to its prolonged production times and the inability to achieve the desired thinness and stability for our experiments, SLS production with PA12 was not further pursued.

### Implantation Methodology

During surgery, the applied techniques did not exceed techniques of vitreoretinal and ocular surgery.^15^ In previous experiments with VLARS and OPTO-EPIRET, the implantation relied on large scleral or corneal incisions and, in some cases, the use of an implantation cone, followed by positioning on the retinal surface and fixation with a retinal tack.^15^^,^^17^ Our group showed that the folded VLARS device could be inserted through a 5.0 to 6.0 mm corneal incision, initially using an implantation cone.^1^^,^^15^ Due to the larger corneal incision, corneal edema and scarring caused an opacity in several cases, making the post-surgery observation more difficult. Overall, during surgery in rabbits, potential risks included mechanical damage to delicate electronic structures during insertion, as well as corneal scarring and/or retinal detachment. Additionally, potential movement or displacement of the VLARS and OPTO-EPIRET implant after surgery could be harmful to the retina.^15^^,^^17^ To this day, comparatively small epiretinal arrays have been implanted successfully in humans.^28^^,^^29^ With increasing size, an increase in risks for, that is, retinal tears and detachment, hypotonia with consecutive hemorrhage, epiretinal gliosis, or inflammation is expected.^15^^,^^17^ These adverse events jeopardize the success of the procedure. Approaching this work, we postulated that the prolonged hypotonia and uncontrolled intraocular flow are major risks for acute retinal damage. The 3D-PLAPS minimized intraocular flow and abbreviates hypotonia periods, leading to a safer and more versatile implantation surgery. We postulate that without the use of 3D-PLAPS, the rate of intraocular lesions at the implantation site will occur more often and might cause significant postoperative complications.

Recently introduced by Ferlauto et al., POLYRETINA, a flexible epiretinal stimulator consisting of a poly(dimethylsiloxane) (PDMS)-photovoltaic interface with a diameter of 12.7 mm, was inserted in a folded state over a scleral incision of about 6.5 mm and intraocularly unfolded.^16^^,^^18^ After optimization of the fabrication, the POLYRETINA was manufactured by plasma-bonding a photovoltaic interface onto a curved support, which allows a tighter folding compared to its earlier version without mechanical damage to the photovoltaic pixels.^18^ The currently used insertion method works with an injector, consisting of three components: a bevelled tube, a narrow tube, and a plunger.^18^ Nevertheless, the insertion of especially large and stiff stimulators in the eye poses both technical and surgical challenges, which cannot be solved by POLYRETINA's injector. Its insertion method relies on the foldability of the epiretinal array, which is not feasible for stiff devices, that is, the OPTO-EPIRET.^17^ Additionally, the alignment to the posterior retinal pole is crucial for implantation of (epi)retinal stimulators. For the implantation of the VLARS, Waschkowski et al. performed thermal contact treatment resulting in an inherent curvature, which was modified by changing the temperature, time of exposure, thickness of the coating, and predetermined intrinsic contraction.^1^ VLARS, and later OPTO-EPIRET, both star-shaped arrays with electrodes on its wings, allow alignment to the posterior pole due to their star shaped design.^15^^,^^17^ Another approach to improve alignment is taken by the POLYRETINA array.^16^ It unfolds intraocularly and aligns to the posterior pole.^16^ Although the use of 3D-PLAPS itself does not directly benefit the epiretinal alignment, we argue that it would not interfere with implantation of the presented preshaped stimulating arrays.

Whereas the fixation of epiretinal stimulators requires retinal tacks,^29^^,^^30^ the 3D-PLAPS needed to address the size and shape of said tacks. We conducted ex vivo experiments regarding the passthrough of retinal tacks, as used for VLARS and OPTO-EPIRET, and found it to be suitable.

In the past, different approaches of implantation and intraocular fixation or retinal arrays have been presented. For epiretinal devices, that is, ARGUS II or IMIE 256, the use of the 3D-PLAPS during implantation is possible. Here, the design of the 3D-PLAPS must be adapted to the array, and its cables and connectors. For the implantation of the ARGUS II device, a temporal sclerotomy of about 5.0 mm was performed.^31^ As shown in this work, customization and production of the 3D-PLAPS is very feasible. For systems not relying on an extraocular cable connection, that is, the POLYRETINA, the application is evident. Currently, a new photovoltaic wireless wide field epiretinal prosthesis is being presented by Schulz et al. in an in vitro and ex vivo model.^32^ With an array size diameter of 14.0 mm, a thickness of 35.0 µm, and overall flexibility, implantation using the 3D-PLAPS seems possible. In the past, alternative methods of intraocular fixation were introduced. The NR600 system (Nano Retina, Herzliya, Israel) used an intraocular lens-fixated helical spring to position the array on the retinal surface.^33^ This unique approach shared similarities with the EPI-RET 3 device invented by members of this group. Similarly, the NR600 is completely intraocularly.^33^ Here, the 3D-PLAPS is limited in its use for implantation, due to the dimensions of the system. Scarcity of recent publications suggest that the project is currently discontinued. With the Intelligent Retinal Implants System (IRIS-2, Pixium Vision S.A., Paris, France), using a novel, image processing unit, another epiretinal stimulating array was unfortunately discontinued in 2018,^34^ as Pixium Vision focused on the Photovoltaic Subretinal Prosthesis (PRIMA, Pixium Visium S.A., Paris, France). Altogether, the 3D-PLAPS is suitable for implanting unfolded arrays with a diameter of 7.0 mm. Establishing the 3D-PLAPS in the implantation of various retinal stimulating systems could reduce risks and improve implantation success.

The 3D-PLAPS was implanted non-permanently. A permanent implantation would bear the risk of infection, inflammation, fluctuations in IOP, and conjunctival erosion with subsequent exposition. Whereas permanent paths into the intraocular space created by implants are used in refractive glaucoma (Ahmed Glaucoma Valve; New World Medical, Inc., Rancho Cucamonga, CA, USA^35^^,^^36^ and Baerveldt Glaucoma Implants; Johnson & Johnson Vision, Santa Ana, CA, USA^35^^,^^36^), and more recently in an effort to prolong intravitreal anti-VEGF release (Port Delivery System, PDS, Susvimo; Genetech, South San Francisco, CA, USA^37^), major risks, that is, increased risk for endophthalmitis, retinal detachment, and suprachoroidal hemorrhage have been reported.^35^^–^^37^ Thus, we argue that chronic implantation of the 3D-PLAPS does not bring benefits outweighing the risks of a permanent path to the intraocular space. In future applications, a chronic implantation could be helpful, although currently not prioritized in our investigation.

All experiments conducted on rabbit and porcine eyes were feasible and reproducible, despite occurring problems as described above. We expect that, in in vivo experiments, significant corneal opacity will not occur prior to surgery. Several cases of retinal detachment were observed in porcine eye experiments before the surgery started. Detachment of the retina in enucleated eyes due to a postmortem break-down of adhesive forces is the most likely cause. In contrast, retinal detachment was not observed in the conducted experiments in postmortem rabbit eyes. The major difficulty in rabbit surgery was the small globe size resulting in a narrow surgical field to insert three trocars and the 3D-PLAPS. A risk when implanting arrays is severe hypotony caused by a large scleral incision. Without the 3D-PLAPS, the incision remains open until the epiretinal stimulator is fixated. Using the 3D-PLAPS shortened the duration of an open incision. The closing plug ensures complete and immediate closure of the system reducing hypotonia, intraocular turbulations, and associated adverse events, that is, hemorrhage, retinal tears, and detachment. However, intraocular hemorrhage from the incision remains a risk. The 3D-PLAPS was designed and printed with rounded edges reducing the possibility of intraocular damage directly caused by the device. Overall, we postulate that the presented methodology can safely be the transferred to in vivo surgeries.

### Pressure Measurements

The objective was to measure IOP fluctuations under various conditions after implanting the 3D-PLAPS device. Additionally, the objective was to examine the IOP in the absence of infusion, given that there are instances during surgery when the infusion might be turned off, that is, adding heavy liquids or maneuvering the epiretinal array. As IOP measurements showed, high IOPs with a closed 3D-PLAPS and a running infusion can be achieved. With non-observable leakage, and stable IOPs, we deduct that intraocular turbulations and severe hypotonia after successful implantation of the 3D-PLAPS were reduced to a minimum. The closing plug allows for closure without the need for additional sutures. Without the possibility to temporally close the scleral aperture, and under a running infusion, intraocular turbulations result in uncontrolled dislocation of the implanted array and possible retinal tearing and detachment. To reduce leakage, the duration of an opened system should be kept as short as possible. Although the risk of hypotension and associated complications were reduced by the 3D-PLASP, they will remain an obstacle to overcome. We propose that these findings require further examination in vivo, including consideration of the potential risks associated with postoperative hypotonia and IOP fluctuations.

## Conclusions

The design, 3D-printed fabrication of the 3D-PLAPS implantation device, and promising observations in surgical handling were crucial steps in the realization of a more feasible methodology to implant large structures, for example, retinal stimulators into human eyes. The 3D-PLAPS can be individually customized to the demands of the given surgery. It has been successfully demonstrated that the 3D-PLAPS can be implanted and explanted without threatening the structural integrity of the eye. Further in vivo experiments are recommended.
